# Supplementary material for: From sequence to enzyme mechanism using multi-label machine learning
Source: BMC Bioinformatics. 2014 May 19;15:150. doi: 10.1186/1471-2105-15-150 (PMC4229970; doi:10.1186/1471-2105-15-150)
Supplement: Additional file 2 — Java code of ml2db. Additional file ml2db_code.tar.gz contains the Java source code to run the multi-label machine learning experiments and save the results to database. The code’s Javadoc is included. [file 1471-2105-15-150-S2.zip › additional file 2/ml2db/ml2db/doc/main/ml2db/mulan/measuremetrics/AnnotationsReader.html]

AnnotationsReader


JavaScript is disabled on your browser.


- Overview
- Package
- Class
- Use
- Tree
- Deprecated
- Index
- Help

- Prev Class
- Next Class

- Frames
- No Frames

- All Classes

- Summary:
- Nested |
- Field |
- Constr |
- Method

- Detail:
- Field |
- Constr |
- Method


main.ml2db.mulan.measuremetrics

## Class AnnotationsReader

- java.lang.Object
- - main.ml2db.mulan.measuremetrics.AnnotationsReader

- ---

    

  ```
  public class AnnotationsReader
  extends java.lang.Object
  ```

  Two sets of [instance, annotation] couples are taken as input, one containing
  "predictions" and another containing the "truth" . The lists must have the
  same instances, but they may have different instance-label assignments.
  Each couple in a list must be in the form [instance identifier, label]. The
  label cannot be null or an empty string. All labels from both lists are
  included into a vector of all possible labels. Each instance is then assigned
  one "predicted" and one "truth" bipartition of valid/nonvalid (true/false)
  labels per each instance (in short, the vector of all labels represented as
  true or false depending if the label was assigned as prediction or as truth)
  . The boolean bipartition may contain all false values (no labels). The
  "truth" and "predicted" bipartitions can then be compared to produce counts
  of true positives, false positives, true negative, false negatives needed to
  calculate machine learning metrics such as accuracy, precision, recall etc.
  Empty instances or class labels should not be provides as they cause the
  program to terminate.

  Version:
  :   3 Dec 2011

  Author:
  :   Luna De Ferrari ldf(at)st-andrews.ac.uk

- - ### Constructor Summary

    Constructors

    Constructor and Description

    `AnnotationsReader(java.lang.String instancesFilePath,
    java.lang.String predictionsFilePath,
    java.lang.String truthFilePath,
    java.lang.String regexpOfSeparator)`
  - ### Method Summary

    Methods

    | Modifier and Type | Method and Description |
    | --- | --- |
    | `java.lang.String` | `bipartitionsToString(java.util.TreeMap<java.lang.String,boolean[]> bipartitions)` |
    | `java.util.TreeSet<java.lang.String>` | `getInstances()` |
    | `java.util.TreeSet<java.lang.String>` | `getLabels()` |
    | `java.lang.String[]` | `getLabelsArray()` |
    | `int` | `getNumberOfInstances()` |
    | `int` | `getNumberOfLabels()` |
    | `java.util.TreeMap<java.lang.String,boolean[]>` | `getPredictedBipartitions()` |
    | `mulan.classifier.MultiLabelOutput` | `getPredictedMultiLabelOutput(java.lang.String instance)` Get the predicted labels for that instance in MultiLabelOutput format |
    | `java.lang.String` | `getPredictionsFilePath()` |
    | `uk.ac.standrews.utils.main.maputils.OneToManyMap<java.lang.String,java.lang.String>` | `getPredictionsInstancesAndLabels()` |
    | `boolean[]` | `getTruthBipartition(java.lang.String instance)` |
    | `java.util.TreeMap<java.lang.String,boolean[]>` | `getTruthBipartitions()` |
    | `java.lang.String` | `getTruthFilePath()` |
    | `uk.ac.standrews.utils.main.maputils.OneToManyMap<java.lang.String,java.lang.String>` | `getTruthInstancesAndLabels()` |

    - ### Methods inherited from class java.lang.Object

      `equals, getClass, hashCode, notify, notifyAll, toString, wait, wait, wait`

- - ### Constructor Detail


    - #### AnnotationsReader

      ```
      public AnnotationsReader(java.lang.String instancesFilePath,
                       java.lang.String predictionsFilePath,
                       java.lang.String truthFilePath,
                       java.lang.String regexpOfSeparator)
      ```

      Parameters:
      :   `instancesFilePath` - the list of instances
      :   `predictionsFilePath` - the list of predicted labels (for the instances that have
          predicted labels)
      :   `truthFilePath` - the list of true labels (for the instances that have true
          labels)
      :   `regexpOfSeparator` -
  - ### Method Detail


    - #### bipartitionsToString

      ```
      public java.lang.String bipartitionsToString(java.util.TreeMap<java.lang.String,boolean[]> bipartitions)
      ```


    - #### getInstances

      ```
      public java.util.TreeSet<java.lang.String> getInstances()
      ```

      Returns:
      :   the instances


    - #### getLabels

      ```
      public java.util.TreeSet<java.lang.String> getLabels()
      ```

      Returns:
      :   the labels


    - #### getLabelsArray

      ```
      public java.lang.String[] getLabelsArray()
      ```

      Returns:
      :   m\_labelsArray


    - #### getNumberOfInstances

      ```
      public int getNumberOfInstances()
      ```

      Returns:


    - #### getNumberOfLabels

      ```
      public int getNumberOfLabels()
      ```

      Returns:


    - #### getPredictedBipartitions

      ```
      public java.util.TreeMap<java.lang.String,boolean[]> getPredictedBipartitions()
      ```

      Returns:
      :   the predictedBipartitions


    - #### getPredictedMultiLabelOutput

      ```
      public mulan.classifier.MultiLabelOutput getPredictedMultiLabelOutput(java.lang.String instance)
      ```

      Get the predicted labels for that instance in MultiLabelOutput format

      Parameters:
      :   `instance` -

      Returns:


    - #### getPredictionsFilePath

      ```
      public java.lang.String getPredictionsFilePath()
      ```

      Returns:
      :   m\_predictionsFilePath


    - #### getPredictionsInstancesAndLabels

      ```
      public uk.ac.standrews.utils.main.maputils.OneToManyMap<java.lang.String,java.lang.String> getPredictionsInstancesAndLabels()
      ```

      Returns:
      :   the predictionsInstancesAndLabels


    - #### getTruthBipartition

      ```
      public boolean[] getTruthBipartition(java.lang.String instance)
      ```

      Returns:


    - #### getTruthBipartitions

      ```
      public java.util.TreeMap<java.lang.String,boolean[]> getTruthBipartitions()
      ```

      Returns:
      :   the truthBipartitions


    - #### getTruthFilePath

      ```
      public java.lang.String getTruthFilePath()
      ```

      Returns:


    - #### getTruthInstancesAndLabels

      ```
      public uk.ac.standrews.utils.main.maputils.OneToManyMap<java.lang.String,java.lang.String> getTruthInstancesAndLabels()
      ```

      Returns:
      :   the truthInstancesAndLabels


- Overview
- Package
- Class
- Use
- Tree
- Deprecated
- Index
- Help

- Prev Class
- Next Class

- Frames
- No Frames

- All Classes

- Summary:
- Nested |
- Field |
- Constr |
- Method

- Detail:
- Field |
- Constr |
- Method
